# Cytotoxicity Evaluation of Anatase and Rutile TiO_2_ Thin Films on CHO-K1 Cells in Vitro

**DOI:** 10.3390/ma9080619

**Published:** 2016-07-26

**Authors:** Blanca Cervantes, Francisco López-Huerta, Rosario Vega, Julián Hernández-Torres, Leandro García-González, Emilio Salceda, Agustín L. Herrera-May, Enrique Soto

**Affiliations:** 1 Instituto de Fisiología, Benemérita Universidad Autónoma de Puebla, 14 sur 6301, Col. San Manuel, 72570 Puebla, Mexico; blanca.cervantes@gmail.com (B.C.); axolotl_56@yahoo.com.mx (R.V.); emilio.salceda@gmail.com (E.S.); 2 Instituto de Investigaciones Biomédicas “Alberto Sols”, Consejo Superior de Investigaciones Científicas-Universidad Autónoma de Madrid, Arturo Duperier, 4, 28029 Madrid, Spain; 3 Facultad de Ingeniería, Universidad Veracruzana, Calzada Ruiz Cortines 455, Boca del Río, 94294 Veracruz, Mexico; frlopez@uv.mx; 4 Centro de Investigación en Micro y Nanotecnología, Calzada Ruiz Cortines 455, Boca del Río, 94294 Veracruz, Mexico; julihernandez@uv.mx (J.H.-T.); leagarcia@uv.mx (L.G.-G.); leherrera@uv.mx (A.L.H.-M.)

**Keywords:** biocompatibility, sensors, cytotoxicity, titanium, titanium dioxide, MTT

## Abstract

Cytotoxicity of titanium dioxide (TiO_2_) thin films on Chinese hamster ovary (CHO-K1) cells was evaluated after 24, 48 and 72 h of culture. The TiO_2_ thin films were deposited using direct current magnetron sputtering. These films were post-deposition annealed at different temperatures (300, 500 and 800 °C) toward the anatase to rutile phase transformation. The root-mean-square (RMS) surface roughness of TiO_2_ films went from 2.8 to 8.08 nm when the annealing temperature was increased from 300 to 800 °C. Field emission scanning electron microscopy (FESEM) results showed that the TiO_2_ films’ thickness values fell within the nanometer range (290–310 nm). Based on the results of the tetrazolium dye and trypan blue assays, we found that TiO_2_ thin films showed no cytotoxicity after the aforementioned culture times at which cell viability was greater than 98%. Independently of the annealing temperature of the TiO_2_ thin films, the number of CHO-K1 cells on the control substrate and on all TiO_2_ thin films was greater after 48 or 72 h than it was after 24 h; the highest cell survival rate was observed in TiO_2_ films annealed at 800 °C. These results indicate that TiO_2_ thin films do not affect mitochondrial function and proliferation of CHO-K1 cells, and back up the use of TiO_2_ thin films in biomedical science.

## 1. Introduction

The applications of titanium dioxide (TiO_2_) films include photocatalysis, photoelectrolysis, and the manufacture of sensors and solar cells. These applications depend on the following characteristics of the TiO_2_ films: specific surface area, crystal and grain size, phase, concentration and dopant. TiO_2_ films can be synthesized through different methods which include sol-gel, hydrothermal, spray pyrolysis, and physical vapor deposition (PVD) [1,2,3,4,5,6]. In health sciences, TiO_2_ is used as a matrix to produce biosensors because of its high conductivity, chemical stability, and good biocompatibility [7]. These sensors can be used in the detection of tumor markers such as the carcinoembryonic antigen and alpha-fetoprotein [8,9,10] as well as in photodynamic therapy for cancer, and in drug delivery systems [11]. Previous studies have shown that the surface of TiO_2_ thin films deposited by direct current magnetron sputtering had good quality, homogeneity, roughness, and biocompatibility. These films were suitable for the culture of functional living neurons that display normal electrical behavior [12]. On account of these findings, we proposed these TiO_2_ thin films to be deposited on the microelectrode surface and the readout circuit of complementary metal oxide semiconductor and micro-electromechanical systems (CMOS-MEMS) for biomedical applications [12,13], for which evaluation of their potential cytotoxicity is required. In order to avoid experimental animal exposure to unjustified risk, studying in vitro cytotoxicity is an essential step prior to the use of TiO_2_ thin films on living animals [14,15]. The cytotoxicity testing of materials is addressed by the International Organization for Standardization 10993 (ISO 10993-5) which presents guidelines to choose suitable tests and define the important principles underlying them [16,17,18]. 

Cytotoxic effects in vitro are evaluated by morphological changes, by analysis of the cell growth rate, or by the study of specific aspects of cellular metabolism [14]. Cells respond rapidly to toxic stress by altering their metabolic and cell growth rates [19]. Therefore, the study of these parameters in cell lines provides valuable information to determine the possible toxic effect of diverse materials. 

The CHO-K1 is a well-established cell line derived from Chinese hamster ovary and considered one of the most sensitive cell lines for cytotoxicity studies [19,20,21]. The tests commonly used to evaluate cytotoxicity are the colorimetric assay with 3-(4,5-dimethylthiazol-2-yl)-2,5-diphenyltetrazolium bromide (MTT), and the trypan blue exclusion assay [22,23]. The MTT test determines the viability and proliferation of cells [15]. MTT is a water-soluble yellow dye which can be reduced to water-insoluble purple formazan crystals through cleavage of the tetrazolium ring by living cells’ mitochondrial succinic dehydrogenase [24]. Formazan is retained in the cells and can be released by solubilization; thus, the concentration of dissolved formazan crystals can be quantified by spectrophotometry, giving a direct measurement of metabolically active living cells. The results are compared to appropriate control samples [2,22,24,25,26,27]. The trypan blue exclusion test is a rapid method to assess cell viability and cell proliferation in response to environmental insults [23]. This test is based on the principle that live (viable) cells do not take up certain dyes, whereas dead (non-viable) cells do because their membrane becomes permeable to the colorant, so analyzing the number of stained as opposed to non-stained cells provides a direct evaluation of the percentage of dead cells in a population and, in addition, the staining aids visualization of the cell morphology [28,29,30]. 

The objectives of this study were to determine the surface morphology, thickness and roughness of the TiO_2_ thin films, and to evaluate the potential in vitro cytotoxicity of the films in crystalline forms (anatase and rutile) on CHO-K1 cells using the MTT and trypan blue assays after 24, 48 and 72 h of culture. 

## 2. Results 

### 2.1. Cytotoxicity Analysis (MTT and Trypan Blue Assays)

**Figure 1:**
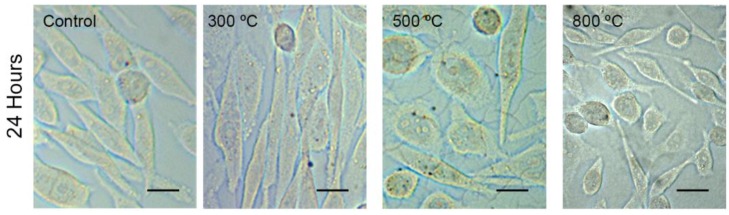
Representative CHO-K1 cells in culture with the negative control condition and in TiO_2_ thin films annealed at 300, 500 and 800 °C. CHO-K1 cells grew with an elongated-ovoid morphology in the control substrate and in all TiO_2_ thin films. The round cells seen in all the images are cells detached from the substrate. Scale bar = 20 µm for all the images.

**Figure 2:**
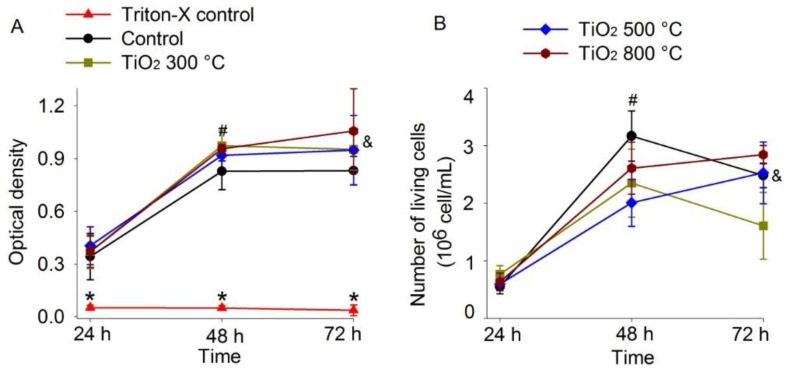
TiO_2_ thin films did not affect cell proliferation of CHO-K1 cells after 48 h. (**A**) Optical density corresponding to Triton-X control (borosilicate glass plus Triton-X), control (borosilicate glass) and TiO_2_ films after 24, 48 and 72 h in culture. The optical density in control substrate and in TiO_2_ films was greater after 48 and 72 h than after 24 h (*p* < 0.05) which indicates cell proliferation; (**B**) Number of unstained CHO-K1 cells (in millions) on control substrate and on TiO_2_ thin films after 24, 48 and 72 h. The number of CHO-K1 cells on the control substrate and TiO_2_ thin films was greater after 48 and 72 h than after 24 h (*p* < 0.05) except for TiO_2_ films annealed at 300 °C. All results are reported as the mean ± standard error and all experiments were performed at least three times. * Triton X-100 24 h vs. Triton X-100 48 or 72 h; ^#^ 24 h vs. 48 h; and & 24 h vs. 72 h.

To assess the cytotoxicity of TiO_2_ thin films, cell viability and cell proliferation on control substrate and on TiO_2_ were determined using the MTT assay and the trypan blue exclusion test. CHO-K1 cells cultured on the control and on the TiO_2_ thin film surfaces (annealed at 300, 500 and 800 °C) showed no ostensive morphological differences (Figure 1). The MTT assay showed that the optical density in TiO_2_ thin film surfaces (annealed at 300, 500 and 800 °C) was not significantly different from that of the control after 24, 48 or 72 h of culture (*p* > 0.05; Figure 2A). As expected, optical density in the Triton-X control was lower than that of the control and the TiO_2_ thin films (*p* < 0.01; Figure 2A), which shows that cells cultured in the Triton-X control did not survive to the application of the detergent Triton-X 1%. The percentage of cell viability on TiO_2_ thin films was similar to that observed on the control substrate after 24, 48 or 72 h of culture (*p* > 0.05). The optical density in the control substrate and in all TiO_2_ films after 48 or 72 h was greater than that after 24 h (*p* < 0.01; Figure 2A), revealing that cell proliferation activity was not influenced by the presence of TiO_2_ thin films. The optical density in the control substrate and in TiO_2_ films after 48 h of culture was not significantly different from the one measured after 72 h (*p* > 0.05). 

**Figure 3:**
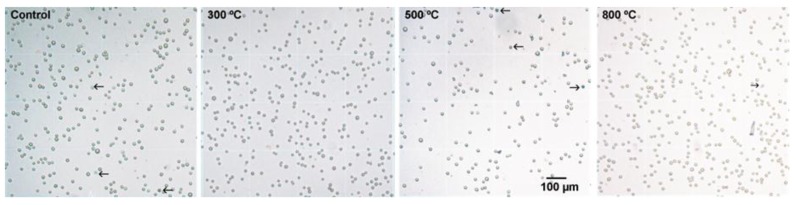
Typical microscope images of cells detached from substrate (control and thin films annealed at 300, 500 and 800 °C after 48 h culture) and stained with trypan blue for analysis of the ratio of living (the cells with an halo and excluding the trypan blue) and dead cells (the cells stained blue) in a Neubauer chamber. The arrows show blue stained cells or lack of blue halo surrounding the cell, indicating its lack of ability to exclude trypan blue. Calibration applies to the four images.

In the trypan blue exclusion test, cell viability on the control (borosilicate glass) and on all TiO_2_ thin films was greater than 98% after 24, 48 and 72 h (Figure 3 and Table 1). The number of viable CHO-K1 cells on TiO_2_ thin films after 24, 48 and 72 h was not significantly different from that on the control substrate (*p* > 0.05; Figure 2B). However, except for TiO_2_ films annealed at 300 °C which were not significantly different (*p* > 0.05), the number of viable CHO-K1 cells on the control substrate and on all TiO_2_ films after 48 or 72 h was greater than that found after 24 h (*p* < 0.01; Figure 2B). The number of viable CHO-K1 cells on the control substrate and on TiO_2_ films after 48 h was not significantly different from the number of cells found after 72 h (*p* > 0.05), indicating that cells did not further proliferate after 48 h of culture, either in control or in TiO_2_ films indicating that normal cell proliferation was not affected by TiO_2_ films.

### 2.2. Surface Roughness of TiO_2_ Thin Films

**Figure 4:**
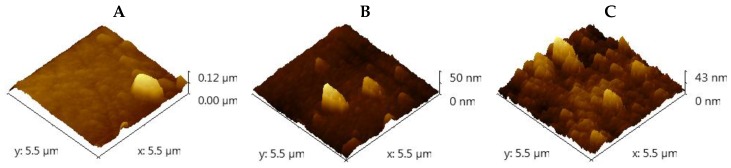
AFM images of the surface of TiO_2_ films deposited by DC magnetron sputtering and annealed at different temperatures: (**A**–**C**: 300, 500 and 800 °C, respectively). The images show that incrementing the annealing temperature produces an increase in the average roughness as a result of the transformation from the anatase to rutile phase.

To characterize the topography of the TiO_2_ films, the samples were analyzed by atomic force microscopy (AFM) (JSPM-5200, JEOL, Tokyo, Japan) in a non-contact mode and processed using Gwyddion software (Gwyddion, Brno, Czech Republic). Figure 4A–C show typical topography three-dimensional (3D) images (5.5 µm × 5.5 µm scan area) of TiO_2_ films annealed at different temperatures. The TiO_2_ films were uniform without voids when crystallized at the 500 °C annealing temperature. The calculated roughness values for the different annealing temperatures are presented in Table 2.

**Figure 5:**
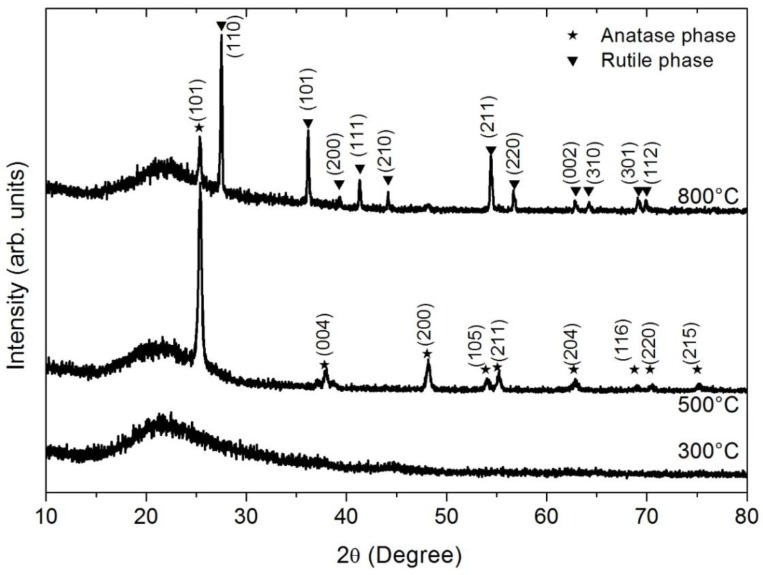
X-ray diffraction patterns for TiO_2_ thin films annealed at different temperatures (300, 500 and 800 °C). X-ray diffraction showed the coexistence of anatase-rutile at 800 °C. The increment in intensity of the rutile phase over the anatase phase was produced by the increase of the thermal annealing treatment.

The increase in the annealing temperature increased the average roughness up to 8.08 nm (Table 2) due to a transformation from the anatase to rutile phase [12,31] as the X-ray diffraction patterns for TiO_2_ thin films annealed at different temperatures show (Figure 5). The X-ray diffraction pattern of the TiO_2_ thin film, post-deposition-annealed at 800 °C, revealed the coexistence of anatase and rutile phases; the intensity of the rutile phase compared to the anatase phase increased as a result of the increment of the thermal annealing treatment. A low RMS value means the TiO_2_ thin film has a dense and homogenous structure. The TiO_2_-anatase phase has a structure that is considerably more homogeneous than that of the TiO_2_-rutile phase [32]. Small clusters of increasing size were produced by heat treatment of temperatures ranging from 300 to 800 °C. 

**Figure 6:**
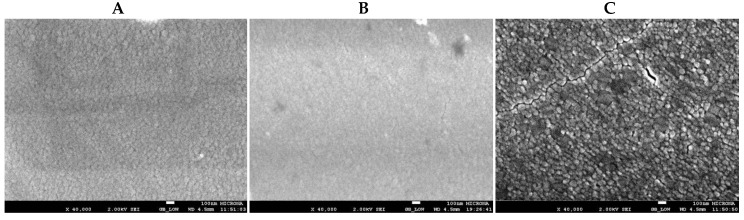
FESEM images (top view) of TiO_2_ thin films annealed at different temperatures: (**A**–**C**: 300, 500 and 800 °C, respectively). Grain size of the TiO_2_ films increased with the rise of the annealing temperature.

The FESEM images were recorded with an acceleration voltage of 2 kV at high vacuum (HV) using a JEOL SEM model JSM-5610LV (Hitachi, Tokyo, Japan). The films were placed in a specimen stub with double-sided adhesive carbon tape, and magnified 40,000 times. Figure 6A–C show typical FESEM micrographs of TiO_2_ thin film surfaces obtained at temperatures ranging from 300 to 800 °C. FESEM measurements of the TiO_2_ thin films were performed both on the surface and on cross-sections. 

**Figure 7:**
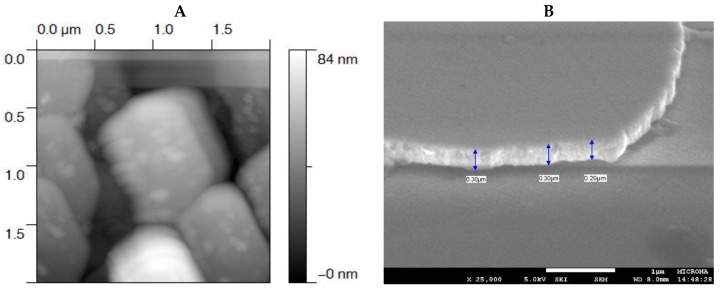
Analysis of TiO_2_ thin films annealed at 300 °C. (**A**) AFM 3D image (top view) of TiO_2_ films (1.5 × 1.5 × 0.084 µm^3^ scan area). The gray scale to the right of the image represents the values on the Z axis: white being the maximum value (84 nm) and black the minimum (0 nm); (**B**) FESEM cross-section of TiO_2_ film recorded with an acceleration of 5 kV at HV and amplified 25,000 times. The image shows that TiO_2_ films had homogenous thickness of about 300 nm.

All TiO_2_ films were uniform, smooth, and composed of small and compact grains on the surface (Figure 6A,B). However, the increase of the temperature during heat treatment caused the formation of clusters approaching a few hundred nanometers in size (Figure 6C), which coincides with the results of AFM (Figure 7A). FESEM imaging of a cross-section of the TiO_2_ films shows that their thickness had values around 300 nm (Figure 7B).

## 3. Discussion

Titanium dioxide is widely used in medical applications due to its excellent biocompatibility and good mechanical strength [6]. Crystalline TiO_2_ occurs in three phases: anatase, rutile, and brookite. Both anatase and rutile have the capability to form bioactive hydroxyl apatite layers in vitro and have good biocompatibility [6]. As a result of its compatibility, the rutile and anatase TiO_2_ surfaces can serve as substrates for growing different cell types [2,33,34,35,36]. Neurons from the mammalian central nervous system (CNS) have a good survival rate on TiO_2_ film surfaces for up to 10 days in culture; rutile surfaces offer good adherence and axonal growth of cultured rat cortical neurons [34]. Moreover, it has also been reported that hepatocytes proliferate and maintain their metabolic activity in long-term culture on rutile and anatase TiO_2_ [2,36,37]. 

We assessed the physical properties and the possible cytotoxic effect of TiO_2_ thin films in their crystalline forms, anatase and anatase/rutile, using CHO-K1 cells that were maintained in culture for 24, 48 or 72 h on TiO_2_ thin film surfaces. The MTT and trypan blue assays indicated that CHO-K1 cells grew equally well on TiO_2_ thin films as on the control substrate, pointing out that the TiO_2_ thin films did not affect cell viability or proliferation. In addition, these cells were viable and functionally similar to those grown on the control substrate. The MTT assay demonstrated they had normal mitochondrial function. These results are consistent with previous data where dorsal root ganglion neurons from the rat were maintained in culture for 18 and 24 h on TiO_2_ thin films retaining their normal electrophysiological properties, proving they were viable and functionally similar to those grown on the control substrate [12]. In contrast to neuronal cultures where cells do not reproduce, the use of CHO-K1 cells added information about the proliferation and metabolic capabilities of living cells on TiO_2_ films.

Cell viability on TiO_2_ thin films was similar to that on the control substrate after 24, 48 or 72 h. The cell count and optical density on the control substrate and on all TiO_2_ films after 24 h were significantly lower than those after 48 and 72 h, which shows cell proliferation in these cultures. However, the cell number and optical density after 48 h were similar to those found at 72 h. This indicates that cells grow steadily until they occupy all the available growth surface; after 48 h in culture, they stop their proliferation both on the control substrate and on TiO_2_ thin films. 

After cells are seeded it takes them around 12–24 h to recover from trypsinization (i.e., reconstruct their cytoskeleton, secrete matrix to aid attachment, and spread out on the substrate) which enables them to reenter the cell cycle. Later on, cells enter their proliferative phase which ends when all the growth surface is occupied or the culture medium exhausted [38]. This explains the lack of increase in cell number after 48 and 72 h in culture. However, our results have shown that TiO_2_ thin films were not cytotoxic in culture even after 72 h. 

Increased cellular proliferation, adhesion and greater efficiency in promoting apatite formation were observed in osteoblasts cultured on TiO_2_ nanotubes annealed at 600 °C in contrast to those grown on nanotubes annealed at other temperatures. The results indicated that tubes annealed to a mixture of anatase and rutile were clearly more efficient than those in their amorphous or plain anatase state [39]. It has been suggested that under this condition, TiO_2_ nanotubes promoted greater cell adhesion and cell proliferation due to their crystalline structure and its morphology, and this would have a common influence on the apatite growth, thereby improving the bioactivity of TiO_2_ nanotubes annealed at 600 °C [39]. In our results cell cultures grown on TiO_2_ thin films annealed at 800 °C produced higher optical density and a larger number of living cells after 72 h, which suggests that at an annealing temperature of 800 °C, the changes in surface morphology and the ratio of anatase to rutile on the TiO_2_ thin films are optimal, among the conditions tested, for the viability and proliferation of CHO-K1 cells.

## 4. Materials and Methods 

### 4.1. TiO_2_ Thin Films

TiO_2_ thin films were deposited on a quartz substrate at room temperature by direct current (DC) magnetron sputtering using a titanium target with a diameter of 50.8 mm. A TiO_2_ ceramic material was located on 20% of the titanium target surface; both materials with a purity of 99.99%. The quartz substrate was cleaned in an ultrasonic bath of acetone (C_3_H_6_O), ethanol (C_2_H_6_O), and distilled water during 5 min at room temperature; this procedure was repeated four times. The TiO_2_ thin film deposition was made under an Argon (Ar) atmosphere and a chamber pressure of 7.46 × 10^−6^ mBar. Argon flow was kept to 15 standard cubic centimeters per minute (sccm) during the TiO_2_ deposition by DC magnetron sputtering. The power supply and substrate temperature were controlled to 100 W and 25 °C, respectively. The TiO_2_ films were then subjected to thermal-annealing treatment to achieve their phase transformation. For this, a thermo scientific thermolyne muffle furnace (model F48025-60-80, Thermo Fisher Scientific Inc., Waltham, MA, USA) was used to keep the temperature constant for one h in each heat treatment. The duration of each heat treatment was lower than that reported elsewhere [40], yet it was enough to reach the required recrystallization and transformation phases. Lastly the TiO_2_ thin films were post-deposition annealed at different temperatures (300, 500 and 800 °C) to the anatase to rutile phase transformation.

The physical properties of the thin films, including film thickness and phase structure, strongly depend on the deposition technique and growth parameters. Therefore, the dependence of the surface morphology and cross-section formation of the TiO_2_ thin films, prepared with different annealing temperatures, were analyzed by Field Emission Scanning Electron Microscopy (FESEM) and Atomic Force Microscopy (AFM).

### 4.2. Cell Culture 

The substrates for cell culture (TiO_2_ films and control glass) were rinsed with deionized water and dried on flat paper towels in a laminar flow hood for 30 minutes. Once dry, the substrates were sterilized by UV light irradiation during 20 minutes. CHO-K1 cells were seeded on the substrates and cultured in Dulbecco’s Modified Eagle's medium (DMEM) supplemented with 10% Fetal Bovine Serum (FBS), 1% L-glutamine, 1% Pyruvate, and 1% penicillin/streptomycin (all of these substances were purchased from Gibco, Thermo Fisher Scientific Inc., Waltham, MA, USA). The cells plated on these substrates were incubated in 55 cm^2^ culture dishes (Sigma-Aldrich, St. Louis, MO, USA) in a humidified incubator at 37 °C with 95% air and 5% CO_2_. The cells were grown to 80% confluence and dissociated with 2.5% trypsin (Gibco) at 37 °C for 2 min to obtain complete cell detachment. Then, 4 mL of culture medium, supplemented with FBS to inactivate the trypsin, was added. The cell suspension was centrifuged at 1500 revolutions per minute (rpm) for 5 min; after this, the supernatant culture medium was removed and the cell pellet was suspended with 4 mL of fresh culture medium [41,42,43]. Finally, about 1 × 10^6^ cell/mL of cell suspension were plated on control (standard borosilicate coverslip), Triton-X control (borosilicate coverslip plus 1% Triton X) and various TiO_2_ thin films. These cells were incubated for 24, 48 or 72 h in an atmosphere of 95% air and 5% CO_2_ at 37 °C before assay. 

### 4.3. MTT Cytotoxicity Assay 

Following incubation, the culture medium was renewed and the cells were incubated with 0.5 mg/mL MTT (Sigma–Aldrich, St. Louis, MO, USA) for 4 h in an atmosphere of 95% air and 5% CO_2_ at 37 °C. After this time, 85% of the culture media (1.7 mL) was replaced with dimethyl sulfoxide (DMSO) (Sigma-Aldrich) 1.7 mL in each well; this procedure destroys the cells and releases the formazan derived from MTT. The concentration of dissolved formazan crystals was spectrophotometrically quantified in a microplate reader at a wavelength of 570 nm (Epoch Microplate Spectrophotometer, BioTek, and Winooski, VT, USA). All experiments were done for at least three times and results expressed as the mean optical density ± standard error. The surviving fraction of cells was calculated for each assay as the percentage of cell viability = (optical density test sample) / (optical density control sample) × 100 [23,43,44]. One-way ANOVA and Duncan's multiple range post-test were used for all comparisons between the control, Triton-X control and TiO_2_ thin films, considering as significant a *p* < 0.05 (although *P*-values lower than 0.01 were included since it means a larger statistical significance level). 

### 4.4. Trypan Blue Exclusion Assay

After cell incubation, the cell pellet was suspended in 200 µL of PBS from which 100 µL were obtained and placed in 100 µL trypan blue 0.4% (Sigma-Aldrich) solution for staining. To determine the cell number on the control substrate and on the thin films, both sides of an hemocytometer were loaded with 10 μL of the suspension and four corners and the middle squares of each side counted. The number of stained and unstained cells was determined. The unstained cell count was taken as a measure of viable cells. Given that each square of the hemacytometer has a surface area of 1 mm^2^ and a depth of 0.1 mm, its volume is 0.1 mm^3^. Since 1 cm^3^ is approximately equal to 1 mL, the cell concentration/mL is the average count per square ×10^4^. The number of living and dead cells was counted on a Leica DM1000 light microscope (Leica Microsystems Inc., Wetzlar, Germany) using digital images obtained with a digital camera ProgRes C10 plus (Jenoptik, Thuringia, Germany) and the associated software ProgRes Capture Pro 2.1 (Jenoptik, Jena, Germany). 

All the experiments were performed in three independent series, and each figure represents data from 10 independent counts from different samples. The percentage of cell viability was estimated as unstained cells/total cells (stained and unstained) × 100 [27]. To compare the results of the control, Triton-X control, and TiO_2_ thin films, one-way ANOVA and Duncan's multiple range post-test were used, considering as significant a *p* < 0.05 (although *P*-values lower than 0.01 were included since it means a larger statistical significance level).

## 5. Conclusions 

These results confirm the feasibility to use TiO_2_ thin films in the crystalline form of anatase and rutile phase as substrates for cell culture. These films allowed the survival and proliferation of CHO-K1 cells. Our results confirmed the in vitro biocompatibility of TiO_2_ thin films, proving that the survival of CHO-K1 cells and dorsal root ganglion neurons was similar; there is also the fact that proliferative and metabolic cell activity were maintained for at least 72 h. Further work will include the study of the biocompatibility of TiO_2_ thin films in vivo, and the study of the mechanical properties and nanoindentation that will explore the possibility of utilizing TiO_2_ thin films on microelectrode surfaces and to include the readout circuit to construct a CMOS-MEMS device that might allow the recording of relevant biological parameters such as micro-potentials caused by pH changes.

## Figures and Tables

**Table 1:** Cell viability in the control and TiO_2_ thin films’ surfaces prepared at temperatures between 300 and 800 °C after 24, 48 or 72 h.

Percentage of Cell Viability	Control	TiO_2_ Films 300 °C	TiO_2_ Films 500 °C	TiO_2_ Films 800 °C
24 h (n = 3)	99.0 ± 0.6	99.2 ± 0.1	99.0 ± 0.5	98.5 ± 0.8
48 h (n = 3)	99.6 ± 0.06	99.5 ± 0.08	99.3 ± 0.06	99.4 ± 0.02
72 h (n = 3)	99.1 ± 0.09	99.0 ± 0.13	99.2 ± 0.12	99.0 ± 0.19

Mean ± standard error.

**Table 2:** Roughness of TiO_2_ thin film obtained from AFM measurements. Columns show the annealing temperature, root-mean square surface roughness (RMS), roughness average (Ra) and area of each sample.

TiO_2_ Films (Temperature)	RMS (nm)	Ra (nm)	Area (μm^2^)
800	8.08	6.67	5 × 5
500	3.66	2.31	5 × 5
300	2.80	2.30	5 × 5

## Abbreviations

The following abbreviations are used in this manuscript:

AFM: Atomic Force Microscopy
CMOS-MEMS: Complementary Metal Oxide Semiconductor and Micro-Electromechanical Systems
CHO-K1: Chinese hamster ovary
DC: direct current
FESEM: Field Emission Scanning Electron Microscopy
MMT: 3-(4, 5-dimethylthiazol-2-yl)-2, 5-diphenyltetrazolium bromide
RPM: revolution per minute
RMS: root-mean square surface roughness
SCCM: standard cubic centimeters per minute
TiO_2_: Titanium Dioxide
